# A Friction Tooth Polish Versus Gritty Tooth Powders and Pastes

**Published:** 1913-05-15

**Authors:** J. P. Carmichael

**Affiliations:** Milwaukee, Wis.


### A FRICTION TOOTH POLISH VERSUS GRITTY TOOTH POWDERS AND PASTES.

BY J. P. CARMICHAEL, D.D.S., MILWAUKEE, WIS.

It is the exception, when we see a clean set of teeth in a human being, and when compared with the mouth of most animals, we readily notice the difference in the luster and cleanliness of the teeth. Why is it that the human teeth should appear so dull and lifeless, even when clean? They are very often half covered over with stains and tartar deposits, and the gums are red and swollen; a glaring example of seeming neglect presenting a condition often associated with pathogenic germ deposits. at the very threshold of our digestive organs; ready to contaminate and pollute the entire system. Our scientists are attributing diseases by the score to infection from unhealthy conditions of the mouth and teeth.

The polish or gloss of enamel was designed by nature to protect the teeth from extraneous deposits and consequent diseases. If this highly glossed surface of the tooth enamel were retained, fermentable matter could not readily adhere, or if the surfaces were always kept polished to the gum margin, the teeth would not decay, nor would there be Dental Pyorrhea.

What then is necessary to keep the enamel of the teeth in a condition so smooth and gleaming, that fermentable substances will not adhere to it, a condition we see in the dog's teeth which are usually clean and shining. Enamel of the teeth is extremely hard, as hard as glass, but when its surface is once marred, or scoured with gritty substances, it is a most difficult problem to so smooth it, as to again restore the luster.

In the primeval days when human beings lived close to nature, the teeth were used for grinding dry coarse foods, and the friction of mastication kept the surfaces polished and healthy; today many of our foods are so prepared as to require little or no mastication. We are well advised of the destructive effects caused by deposits remaining too long upon the teeth, and our patients are easily convinced of the uncleanly condition of the teeth by using a so called disclosing solution. The first formations that adhere to the enamel and destroy its luster, and which may not be visible, can readily be detected by applying this solution of iodine which is painted over the entire crowning of the tooth, and may then be readily washed away from the enamel, leaving the gloss unaffected.

This test clearly reveals that the harmful adhesions first formed upon the teeth about their necks or at the gum margins and between the teeth; they are largely believed to be glutinous plaques mixed with exfoliation of the gum tissue, combined with abnormal secretions which adhere so closely to the enamel that they are not easily, if at all, removed by the tooth brush.

Upon the neglected teeth, these exfoliations adhere, where there is the least friction; we then have a foundation for other deposits, combining food debris and sticky substances that gather in quantities sufficient to undergo septic decomposition. The bacteria multiplying in the decomposition of these deposits is generally accepted as the primary cause of the dissolution of tooth tissue, resulting in caries; and the same microbic action may well be regarded as the primary cause of all forms of peridentitis.

Our usual prescription is the tooth brush and dentifrice which is very beneficial in removing loose particles of food, and to stimulate healthy action to the gums; but it will not remove from the teeth the adhesions mentioned, and which are likely to become most harmful.

Dentists have, for a long time, advised the use of some abrasive substance in the form of tooth powders and pastes applied by means of the brush, that would clean the teeth by a scouring process, but I contend that this is where we have made a grave mistake. If by our treatment we are to prevent disease, then let us begin by restoring the brilliancy of the tooth surface, so that it is possible for the enamel to more easily ward off all foreign deposits.

All the substances in general use for cleaning teeth are harsh and gritty, even though they be of very fine grit, they accomplish the purpose only by a scouring prosess, thus gradually destroying the natural gloss of the enamel. Although these scratches are not visible to the naked eye, they are sufficient to destroy the brilliancy, and leave the surface all the more susceptible to receive foreign adhesions. In other words, the more we scour the teeth, with the ordinary tooth powders the more we must scour to keep them clean, to say nothin of destroying the life luster. As proof of this, it is only necessary to dry the teeth that have been customarily brushed with powders to disclose the fact that the enamel gloss has been dulled.

It may be necessary, sometimes, for the dentist to apply a very finely powdered abrasive, to remove stains in cleaning teeth, but this should not be used even by the dentist over the entire surface of the teeth, but confined to the stained area, and then using a preparation of a character that will not scratch.

Experimentation has proven to me that a frictional dry rub is not only effective in removing the adhesions, but the life luster becomes more intensified. I have prepared a preparation which, when properly used with a friction polish, possesses enough resistance that when applied dry by a rubbing process, it will smooth the surface and bring the real life luster to the enamel. This preparation is made by calcining certain minerals and incorporating them with rare earth nitrates at very high temperatures. By this process of treatment, an impalpable powder is obtained, having peculiar frictional properties specially adapting it for polishing very hard substances without scratching them. Even a porcelain tooth may be polished with it so as to give it a life-like appearance that heretofore has never been attained. I have put this material on the market under the trade name of *Carmi-Lustro.*

The general formula below will give its essential constitution:

℞—Calcined minerals and fine Earths.
Zinc Oxide, astringent.
Betanapthol and Boric Acid, antiseptics,
Aromatic Oils for flavoring.

This preparation is the result of several years of study as to the proper prophylactic treatment of the teeth, and I have applied this principal of polishing the teeth in my practice with notable success for several years since I perfected the above compound. I have also given many clinics and demonstrations among my friends and other practitioners. I have been gratified at the complimentary assertions and favorable reports of other, and believe the preparation has sufficient merit to justify me in placing it in the hands of the profession.

Instruct patients for home treatment to dry the teeth and apply Carmi-Lustro daily with a soft linen cloth, or a cotton roll, rubbing them vigorously. The more constantly this course is followed, the more beautiful and highly polished will the teeth become.

Dentists may also instruct their patients how to tape and polish the neck of the teeth. Inform your patient, also, that after the polish has been restored to the teeth, any gritty tooth powder or other like substance they may apply will presently destroy the luster. Many tooth powders contain powdered pumice, cuttle, fish bone or prepared chalk. Pumice is found in volcanic eruptions, and is hard enough to scratch glass; precipitated chalk, although it may be reduced to an extremely fine powder, being of crystalline formation, has sharp angles and will dull the high polish of the enamel of teeth.

Dr. F. H. Skinner in a paper on prophylaxis says: "It is universally agreed that neither Pyorrhea or decay is found when surfaces have always been kept smooth and polished; that all decay is the result of by-products of bacteria, which can only be removed by friction."

The enamel must be kept so smooth and brilliant that it will ward off disease producing deposits. To accomplish this, we must adopt such measures as will restore the teeth to a state of nature, which is in line with the highest attainment in dentistry and ought to make for preventive dentistry.

